# N-3 polyunsaturated fatty acids decrease levels of doxorubicin-induced reactive oxygen species in cardiomyocytes -- involvement of uncoupling protein UCP2

**DOI:** 10.1186/s12929-014-0101-3

**Published:** 2014-11-18

**Authors:** Hsiu-Ching Hsu, Ching-Yi Chen, Ming-Fong Chen

**Affiliations:** Department of Internal Medicine, National Taiwan University Hospital, 7 Chung-Shan S Rd, Taipei, Taiwan; Department of Animal Science and Technology, National Taiwan University, No. 50, Lane 155, Sec 3, Keelung Rd, Taipei, 10672 Taiwan; Present address: 50, Lane 155, Sec 3, Keelung Rd, Taipei, 106 Taiwan

**Keywords:** EPA, DHA, Doxorubicin, ROS, UCP2

## Abstract

**Background:**

Use of the chemotherapeutic drug doxorubicin (DOX) is associated with serious cardiotoxicity, as it increases levels of reactive oxygen species (ROS). N-3 polyunsaturated fatty acid dietary supplements can be of benefit to patients undergoing cancer therapy. The aims of this study were to determine whether DOX-induced cardiotoxicity is related to mitochondrial uncoupling proteins and whether eicosapentaenoic acid (EPA, C20:5 n-3) or docosahexaenoic acid (DHA, C22:6 n-3) affects DOX-induced cardiomyocyte toxicity.

**Results:**

Treatment of H9C2 cells with DOX resulted in decreased cell viability and UCP2 expression. Treatment with 100 μM EPA or 50 μM DHA for 24 h resulted in a maximal mitochondria concentration of these fatty acids and increased UCP2 expression. Pretreatment with 100 μM EPA or 50 μM DHA prevented the DOX-induced decrease in UCP2 mRNA and protein levels, but these effects were not seen with EPA or DHA and DOX cotreatment. In addition, the DOX-induced increase in ROS production and subsequent mitochondrial membrane potential change (∆ψ) were significantly attenuated by pretreatment with EPA or DHA.

**Conclusion:**

EPA or DHA pre-treatment inhibits the DOX-induced decrease in UCP2 expression, increase in ROS production, and subsequent mitochondrial membrane potential change that contribute to the cardiotoxicity of DOX.

**Electronic supplementary material:**

The online version of this article (doi:10.1186/s12929-014-0101-3) contains supplementary material, which is available to authorized users.

## Background

The anthracycline antibiotic doxorubicin (DOX) is one of the most highly prescribed chemotherapeutic drugs for the treatment of a variety of human cancers [1]. Unfortunately, in addition to its potent antitumor effect, DOX use is associated with a number of unwanted side effects, especially serious cardiac toxicity, and this complication represents a major obstacle to the prolonged use of the drug [2]. DOX cardiotoxicity seems to be a multi-factorial process that leads to cardiomyocyte death as the terminal downstream event. DOX exerts its anticancer and cardiotoxic actions by different mechanisms, the anticancer response being associated with DNA intercalation, topoisomerase-II inhibition, and apoptosis, and the cardiotoxicity being mainly ascribed to oxidative stress [3,4]. The role of oxidative stress in DOX-induced cardiotoxicity is supported by the fact that cardiotoxicity is reduced in animals by treatment with a variety of antioxidants [5,6]. In addition, DOX causes mitochondrial dysfunction in the heart by modulating the mitochondrial membrane lipid content, interacting with cardiolipin, inhibiting the activity of several mitochondrial proteins, and displacing α-enolase from mitochondria [7,8]. A recent study indicated that DOX induces cardiotoxicity mainly by disrupting the catalytic cycle of topoisomerase 2β (Top2β) [4]. Through this disruption of Top2β function, DOX causes DNA double-strand breaks and changes in the transcriptome [4], which contributes to the generation of reactive oxygen species (ROS) and to the mitochondrial dysfunctions, thus resulting in apoptosis.

Several observational and experimental studies have demonstrated the beneficial effects of n-3 poly-unsaturated fatty acids (PUFAs) in cardiovascular disease [9,10]. The results from such studies justify n-3 PUFA supplementation in the primary and secondary prevention of several clinical conditions, including coronary heart disease, sudden cardiac death, and heart failure [9,10]. Consumption of n-3 PUFAs in fish oil, specifically eicosapentaenoic acid (EPA) and docosahexaenoic acid (DHA), decreases the risk of heart failure and attenuates pathological cardiac remodeling in response to pressure overload. Dietary supplementation with EPA and DHA can also affect cardiac mitochondrial function and energetics through the alteration of membrane phospholipids [11]. In addition, the benefits of fish oil dietary supplements given before or during cancer therapy include increasing the anticancer drug efficacy [12-14] and reducing the cardiac side effects of various chemotherapeutic treatments [15,16].

The mitochondrial uncoupling proteins UCP2 and UCP3 have been shown to be important in the fields of thermogenesis, obesity, diabetes, free radical biology, and heart failure [17]. When activated by superoxide, ROS, or alkenals, UCPs increase mitochondrial proton conductance to cause mild uncoupling and so decrease mitochondrial superoxide production, and are thus providing protection against oxidative damage [17]. Nedergaard and Cannon [18] reported that UCP mRNA levels are increased in the presence of long-chain free fatty acids. In addition to regulating the mitochondrial superoxide production, UCP overexpression protects against oxidative stress-induced apoptosis [19].

Previous studies have shown that fish oil dietary supplements are of benefit to patients undergoing cancer therapy [15,16,20]; however, it is still unclear whether EPA or DHA has a beneficial effect on DOX therapy, especially in terms of reducing its cardiotoxicity. Moreover, there have not been any studies on a possible association between DOX cardiotoxicity and UCP2 function. The aim of this study was to determine whether DOX-induced toxicity is related to UCP2 levels and to investigate whether EPA or DHA affects DOX-induced cardiomyocyte toxicity.

## Methods

### Cell culture and treatment

Cardiomyoblast H9C2 was obtained from the American Type Culture Collection (ATCC) and cultured in Dulbecco’s Modified Eagle’s Medium (DMEM; Gibco BRL, Grand Island, NY. USA) containing 10% fetal bovine serum (FBS, Hyclone, Auckland, NZ), 2 mM L-glutamine, 0.1 mM non-essential amino acids (Gibco BRL, Grand Island, NY, USA), 100 units/ml of penicillin, and 100 μg/ml of streptomycin (Gibco BRL, Grand Island, NY, USA) at 37°C in a humidified chamber with 5% CO_2_. After the subcultured cells had been incubated for 24 h and become attached to the plate, various concentrations of EPA or DHA (Sigma-Aldrich, St. Louis, MO, USA) were added in serum-free DMEM containing 0.1% bovine serum albumin (BSA) (Sigma-Aldrich, St. Louis, MO, USA) for 24 or 48 h to investigate the incorporation of n-PUFAs into subcellular fractions. In addition, H9C2 cells with or without pretreatment for 24 h with EPA or DHA in DMEM containing 0.1% BSA were left untreated or were treated with 1 μM DOX in DMEM containing 10% FBS for 24 h, then were harvested for analysis, while other cells were cotreated for 24 h with EPA or DHA plus DOX in DMEM containing 10% FBS.

After DOX treatment, the cells were incubated for 2 h with MTT (3-(4,5-dimethylthiazol-2-yl)-2,5-diphenyltetrazolium bromide; Sigma-Aldrich, St. Louis, MO, USA), then the absorbance of the sample at 450 nm was measured using a microplater reader. Experiments were repeated three times.

### Subcellular fractionation

All steps were performed at 4°C. The cells were lysed with 250 mM sucrose, 20 mM HEPES, 10 mM KCl, 1.5 mM MgCl_2_, 1 mM EDTA, and 1 mM EGTA and the lysate passed through a 25G needle 10 times and left for 20 min, then centrifuged at 720 g for 5 min to obtain the nuclear pellet. The supernatant was removed and centrifuged at 10,000 g for 20 min to obtain the mitochondria fraction, then the supernatant was centrifuged at 100,000 g for 60 min to obtain the membrane and cytosol fractions [21].

### Determination of fatty acid composition

To determine the fatty acid composition, lipids were extracted from the total cell lysate or mitochondria. Each sample plus 50 μg of 1,2-dinonadecanoyl-sn-glycero-3 phosphocholine (C19:0 PC; Avanti Polar Lipids Inc.; Alabaster, Alabama), as an internal standard was homogenized in a 2/1/0.75 (v/v) mixture of methanol/chloroform/water and centrifuged at 1200 g for 30 min at 16°C and the upper phase discarded. The lower phase was dried under nitrogen and resuspended in 14% borontrifluoride methanol (Merck Schuchardt OHG, Hohenbrunn,Germany) followed by heating at 90°C for 40 min. After cooling, the free fatty acids were extracted with an 8/3 (v/v) mixture of pentane/water by vortexing and the organic phase recovered and dried under nitrogen. The extracts was resuspended in 50 μl of heptane for gas chromatography analysis on a capillary column (DB-23 60 m × 0.25 mm × 0.25 μm, Agilent J&W, USA). The gas chromatograph was an Agilent 7890 series GC equipped with a flame ionization detector (FID/EPC G3440A) and an autoinjector module. The oven temperature was held at 120°C for 2 min, then was increased at 8°C per min to 200°C and held at 200°C for 1 min, then was increased by 3°C per min to 240°C and held at 240°C for 2 min. The injector and detector were both at 240°C and the flow rate of carrier gas, helium, air and nitrogen was 40 ml/min, 450 ml/min, and 25 ml/min, respectively. Components were identified by comparison of the retention time with those of authentic standards (Supelco 37 Comp. Fame Mix™, Supelco Inc. Belletonte PA) [22].

### Quantitative real-time PCR analysis

Total RNA was isolated using TRIzol reagent according to the manufacturer’s instructions (Promega Corporation, Madison, WI, USA). RNA samples (2 μg) were reverse transcribed using random hexamer primers and M-MLV reverse transcriptase (Promega Corporation, Madison, WI, USA) and the cDNA used for real-time PCR, performed on a StepOnePlus™ Real-Time PCR Detection System using Power SYBRGreen Master Mix™ (Applied Biosystems Inc. Foster, CA, USA) following the manufacturer’s protocol. The PCR amplification reaction mixture (25 μl) contained 50 ng of cDNA, 12.5 μl of SYBR Green Supermix, and 0.2 μM of the UCP2 or GAPDH-specific primer pair. The optimal primer concentrations were determined in preliminary experiments. The PCR primers were designed using ABI designer software Primer Express version 2.0 (Applied Biosystems Inc. Foster, CA, USA) and their sequences were UCP2 (Genbank accession nos. **AB 010743.1**) forward 5’-GAA AGG CTC TCC CAA TG-3’ and UCP2 reverse 5’-GGA GGT CGT CTG TCA TGA GG-3’ and GAPDH (Genbank accession nos. **NM_017008.4**) forward 5’-GGC CTT CCG TGT TCC TAC C CAG-3’ and GAPDH reverse 5’-CGG CAT GTC AGA TCC ACA AC-3’. In order to confirm amplification specificity, the PCR products from each primer pair were subjected to melting curve analysis. The reaction conditions were incubation at 50°C for 2 min and initial denaturation at 95°C for 10 min, followed by 40 cycles of denaturation at 95°C for 20 s and annealing at 60°C for 1 min. After real-time PCR, the temperature was increased from 60 to 95°C at a rate of 0.5°C per second to construct a melting curve. A negative control without cDNA was run in parallel with each assay. Each reaction mixture was amplified in triplicate and the results calculated based on the ΔΔCt method [23]. The cycle threshold (Ct) value for the UCP2 gene was corrected using the mean Ct value for the GAPDH gene. Relative gene expression was expressed as the fold change (2^−ΔΔCt^) relative to expression in the untreated control.

### Western blotting

Cells were harvested and total cell lysates prepared using lysis buffer [20 mM Tris–HCl (pH 7.2), 2 mM EGTA, 5 mM EDTA, 500 μM sodium orthovanadate, 10 mM sodium fluoride, 1% Triton X-100, 0.1% SDS and protease inhibitor cocktail]. Protein concentrations were determined using protein assay reagents (Bio-Rad, Hercules CA, USA). Forty to sixty micrograms of protein lysate was analyzed by SDS-polyacrylamide gel electrophoresis. After transfer of the proteins from the gel to a nitrocellulose membrane (Amersham Pharmacia Biotech, Freiburg, Germany), the membranes were blocked for 1 h at room temperature in phosphate-buffered saline (PBS) containing 0.05% Tween 20 (PBS-T) and 5% nonfat dry milk, then were incubated with anti-UCP2 (Santa Cruz Bio. Inc., MA USA) or anti-VDAC1(Abcam, London, UK) polyclonal antibodies, followed by horseradish peroxidase-conjugated secondary antibodies (pharMingen, San Diego CA, USA). The immunoreactive bands were visualized using an enhanced chemiluminescence kit (Perkin-Elmer Life Sciences, Boston, MA, USA) and analyzed by QUANTITY ONE (Bio-Rad Hercules CA, USA). β -actin was used as the internal control.

### Intercellular ROS measurement

2′,7′-dichlorodihydrofluorescein diacetate (H_2_DCF-DA; Molecular Probes, Eugene, OR, USA) was used to measure intercellular ROS production. After treatment, 10 μM H_2_DCF-DA was added to the culture medium for 30 min, then the cells were trypsinized and resuspended in Hanks’ balanced salt solution (Gibco, Grand Island, NY, USA). The fluorescence of the dichlorofluorescein formed from the oxidation of H_2_DCF-DA by cellular oxidants was measured using a FACScalibur flow cytometry (BD Bioscience, San Jose, CA, USA) with an excitation wavelength of 488 nm and an emission wavelength of 525 nm. Data were analyzed using WinMDI 2.8 software. As a positive control, 100 μM H_2_O_2_ was added to H9C2 cells 2 h before cell harvesting.

### Mitochondrial membrane potential measurement

Energy released during the oxidation reactions in the mitochondrial respiratory chain is stored as a negative electrochemical gradient across the mitochondrial membrane, and the mitochondrial membrane potential (∆ψ) is referred to as being polarized. A change in the ∆ψ occurs during apoptosis and necrosis (depolarization) and cell cycle arrest (hyperpolarization) [24]. The mitochondrial ∆ψ was measured using a fluorescent cationic dye, JC-1 (5,5’,6,6’-tetrachloro-1,1’,3,3’- -tetraethylbenzimidazolcarbocyanine iodide) provided in a commercial kit (MitoScreen™ Kit, BD Bioscience, San Jose, CA, USA). After treatment, the H9C2 cells were incubated with JC-1 working solution for 15 min at 37°C, then were trypsinized and resuspended for analysis by FACScalibur flow cytometry (BD Bioscience, San Jose, CA, USA). Data were analyzed using WinMDI 2.8 software.

### Statistical analysis

All data are expressed as the mean ± SD. The significance of differences was determined by one-way ANOVA followed by Fisher’s test. Statistical analyses were performed using SAS (version 6.011; SAS Institute Inc, Cary, NC. USA). A p value <0.05 was considered statistically significant.

## Results

### DOX decreases cell viability and UCP2 expression

**Figure 1:**
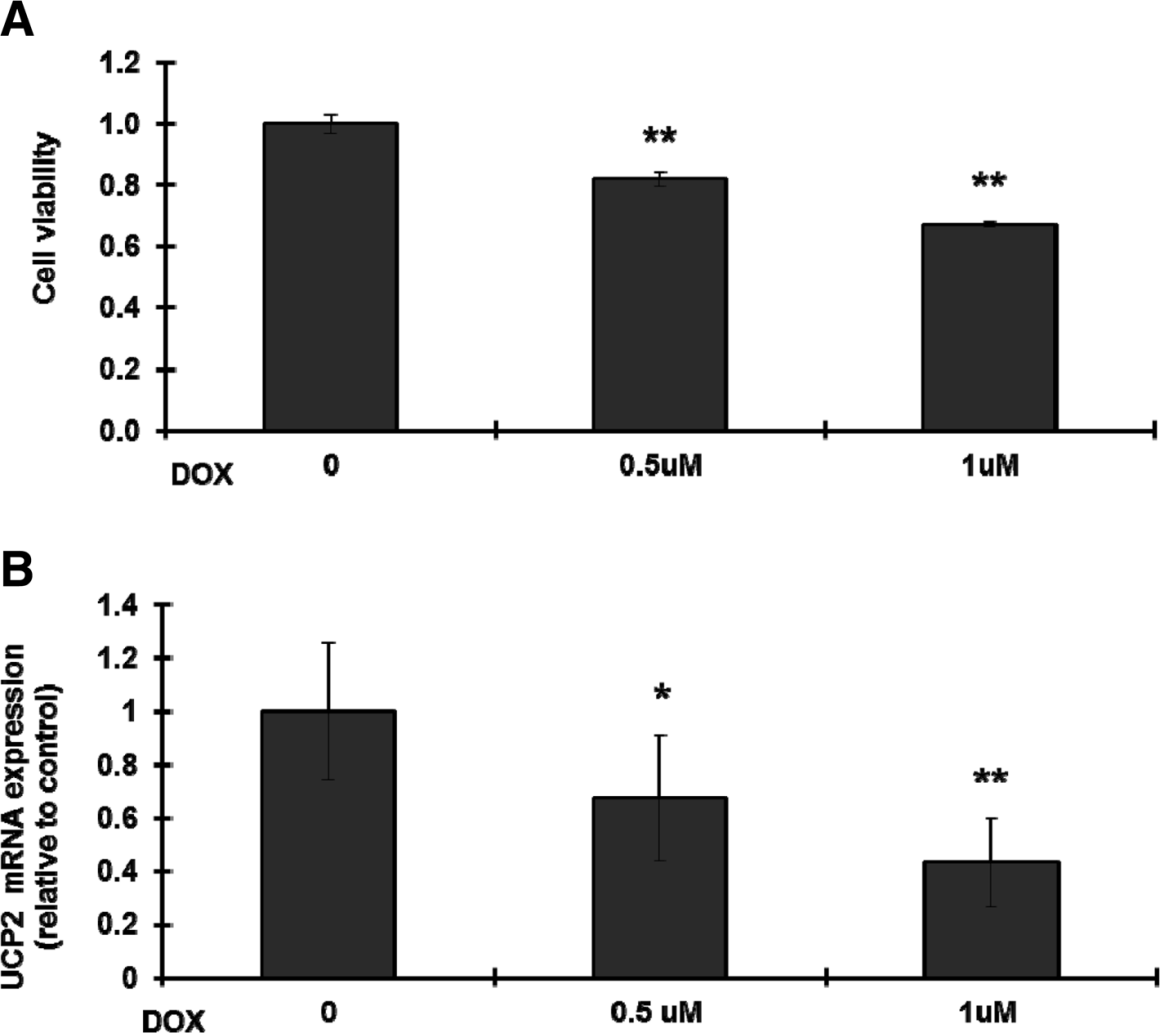
**Doxorubicin decreases cell viability and UCP2 mRNA levels in H9C2 cells.** H9C2 cells were left untreated or were treated with 0.5 or 1 μM doxorubicin (DOX) for 24 h, then **(A)** cell viability was measured by the MTT method and **(B)** UCP2 expression was measured by reverse transcription and quantitative real-time PCR, with GAPDH (glyceraldehyde-3-phosphate dehydrogenase) as the internal control. The results are expressed relative to the control value and are the mean ± S.D. for three independent experiments. *: p <0.05 compared to the untreated control; **: p <0.01 compared to the untreated control.

H9C2 cell viability was significantly decreased after 24 h treatment with 0.5 or 1 μM DOX (Figure 1A). To examine whether this DOX-induced cell cytotoxicity was associated with altered expression of UCP2, mRNA was extracted from DOX-treated H9C2 cells and subjected by reverse transcription and quantitative real-time PCR analysis. As shown in Figure 1B, UCP2 mRNA expression was significantly decreased by treatment with 0.5 or 1 μM DOX. The trends for cell viability and UCP2 expression were similar, suggesting the two are related.

### Fatty acid composition of the cell and mitochondria after EPA or DHA treatment

In order to investigate whether EPA or DHA was protective against the DOX-induced decrease in UCP2 expression in H9C2 cells, we first determined the concentrations of EPA and DHA at which the mitochondria were saturated with the respective fatty acid, then measured UCP2 mRNA levels at these concentrations. Different concentrations of EPA or DHA were added to H9C2 cells for 24 h, then the cells were harvested and separated into subcellular compartments, followed by fatty acid pattern analysis. We found that the EPA or DHA content of the total cell lysate increased as the concentration of EPA or DHA used increased, whereas that in the mitochondria reached a maximum using concentrations of 100 μM EPA or 50 μM DHA (Additional file 1). Table 1 shows that addition of either EPA or DHA resulted in an increase in n-3 PUFAs in the fatty acid composition of the total cell lysate at the expense of a significant decrease in oleic acid (C18:1, n-9) and arachidonic acid (C20:4, n-6), while, in the mitochondria, in addition to the above decreases, there were significant decreases in levels of palmitic acid (C16:0) and stearic acid (C18:0). It is noticeable that the docosapentaenoic acid (C22-5, n-3) content was increased in both the cell lysate and mitochondria after EPA treatment, suggesting that acetyl-CoA elongation of EPA occurs in H9C2 cells, resulting in a marked increase in the n-3/n-6 PUFA ratio in the mitochondrial fatty acid.

**Table 1:** **Mole percentage of fatty acids in H9C2 cell (total cell lysate or mitochondria) after 24 h treatment with 100 μM EPA or 50 μM DHA**

**Fatty acid**	**Total**			**Mitochondria**		
**(Mol %)**	**Control**	**EPA**	**DHA**	**Control**	**EPA**	**DHA**
C12:0	1.16 ± 0.43	1.55 ± 0.32	1.58 ± 0.24	1.25 ± 0.32	1.19 ± 0.25	1.76 ± 0.33
C14:0	0.60 ± 0.06	0.86 ± 0.06	1.05 ± 0.07	0.98 ± 0.02	0.83 ± 0.04	0.72 ± 0.05
C15:0	0.21 ± 0.06	0.22 ± 0.03	0.74 ± 0.32	0.74 ± 0.19	0.75 ± 0.28	0.86 ± 0.39
C16:0	15.22 ± 2.00	15.06 ± 1.98	15.89 ± 1.86	18.94 ± 1.89	15.32 ± 2.11*	15.56 ± 1.54*
C:16:1 t	5.23 ± 1.18	5.16 ± 0.98	3.83 ± 1.18	5.73 ± 1.18	5.63 ± 0.26	5.83 ± 0.98
C16:1	2.86 ± 0.98	1.22 ± 0.81	0.97 ± 0.81	2.65 ± 0.90	2.65 ± 0.60	3.45 ± 1.86
C17:0	1.19 ± 0.23	1.52 ± 0.31	1.99 ± 0.23	1.45 ± 0.32	1.58 ± 0.26	2.49 ± 0.23
C18:0	21.82 ± 3.72	20.15 ± 3.56	19.36 ± 3.92	22.76 ± 1.72	18.73 ± 2.25*	18.36 ± 3.72*
C18:1	14.50 ± 1.06	12.69 ± 1.04*	12.50 ± 1.06*	13.40 ± 1.86	9.04 ± 2.02*	10.95 ± 2.06*
C:18:1 n-7	6.24 ± 0.98	5.06 ± 0.86	5.24 ± 0.95	5.31 ± 0.99	5.78 ± 0.67	5.44 ± 1.80
C18:2 t n-6	2.35 ± 0.46	2.26 ± 0.46	2.35 ± 0.84	3.86 ± 0.86	3.35 ± 0.76	3.35 ± 0.46
C18:2 n-6	2.97 ± 0.43	2.65 ± 0.34	2.40 ± 0.43	2.28 ± 0.58	2.59 ± 0.37	2.40 ± 0.73
C18:3 n-3	0.27 ± 0.17	0.37 ± 0.25	1.26 ± 0.87	0.17 ± 0.04	0.65 ± 0.25	0.67 ± 0.27
C20:0	0.31 ± 0.32	0.56 ± 0.12	0.66 ± 0.32	0.12 ± 0.02	0.18 ± 0.04	0.65 ± 0.32
C20:1	0.37 ± 0.03	0.38 ± 0.04	0.15 ± 0.03	0.24 ± 0.01	0.22 ± 0.02	0.87 ± 0.43
C20:2	1.79 ± 0.31	1.49 ± 0.28	1.15 ± 0.35	1.12 ± 0.05	1.36 ± 0.04	1.15 ± 0.41
C21:0	0.61 ± 0.26	0.51 ± 0.08	0.51 ± 0.26	1.06 ± 0.02	1.52 ± 0.21	1.17 ± 0.06
C20:3 n-6	1.14 ± 0.26	0.97 ± 0.17	1.02 ± 0.21	0.85 ± 0.18	0.93 ± 0.20	0.54 ± 0.06
C20:4 n-6	8.56 ± 1.15	6.67 ± 1.05*	5.59 ± 1.25*	7.67 ± 1.15	5.04 ± 0.19*	4.89 ± 0.25*
C20:3 n-3	0.65 ± 0.15	1.22 ± 0.39	0.90 ± 0.21	0.84 ± 0.19	0.70 ± 0.16	1.44 ± 0.59
C20:5 n-3	0.94 ± 0.25	5.52 ± 1.13*	0.91 ± 0.13	0.72 ± 0.13	5.52 ± 1.23*	0.91 ± 0.13
C23:0	1.14 ± 0.36	0.77 ± 0.56	0.99 ± 0.24	0.32 ± 0.15	0.29 ± 0.12	0.54 ± 0.36
C24:0	0.91 ± 0.13	0.73 ± 0.13	0.84 ± 0.25	0.58 ± 0.15	0.42 ± 0.14	0.99 ± 0.43
C22:5 n-3	3.18 ± 0.88	5.99 ± 1.23*	3.60 ± 0.48	3.05 ± 0.98	10.22 ± 2.08*	3.45 ± 0.96
C22:6 n-3	3.45 ± 0.96	2.97 ± 0.99	13.54 ± 2.89*	3.90 ± 0.56	4.24 ± 0.36	10.65 ± 1.23*
C24:1	1.91 ± 0.12	1.70 ± 0.19	1.54 ± 0.23	0.41 ± 0.11	0.28 ± 0.13	0.51 ± 0.23
n-3/n-6 PUFA	0.56 ± 0.08	1.35 ± 0.14*	1.85 ± 0.19*^△^	0.59 ± 0.09	1.71 ± 0.18*	1.35 ± 0.15*^△^

All data are the mean ± S. D. for three separate experiments. PUFA: polyunsaturated fatty acids. *:p <0.05 as compared to the corresponding control; ^△^:p <0.05 compared to the corresponding EPA treatment.

**Figure 2:**
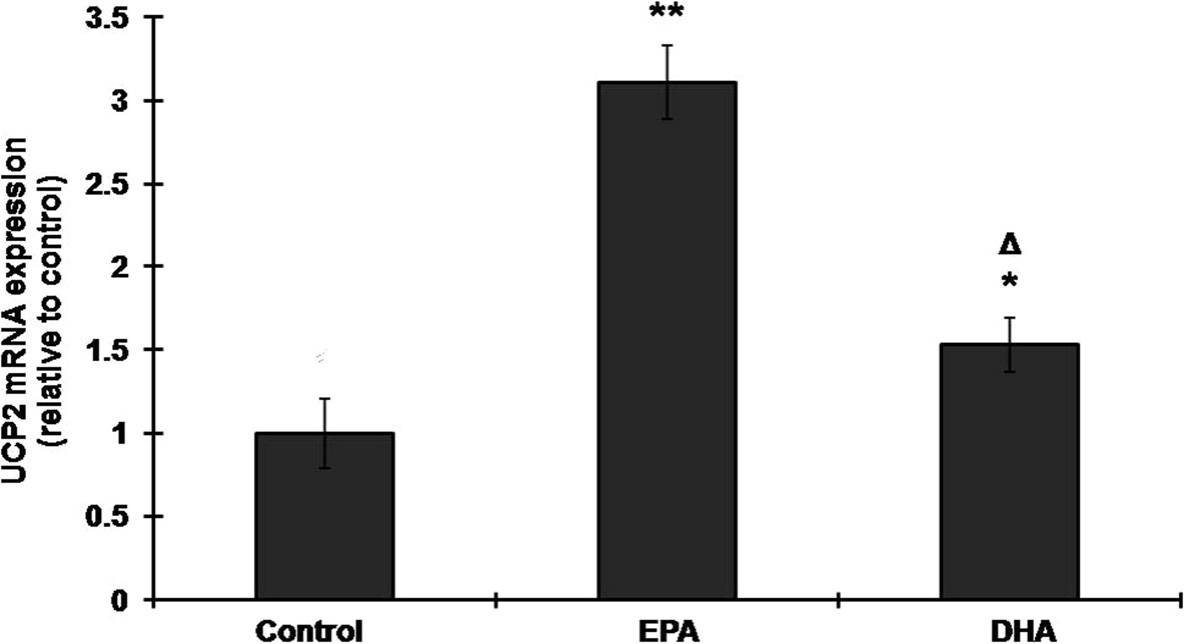
**EPA or DHA increases UCP2 mRNA levels in H9C2 cells.** H9C2 cells were left untreated or were treated with 100 μM EPA or 50 μM DHA for 24 h, then UCP2 mRNA expression was measured by reverse transcription and quantitative real-time PCR, with GAPDH (glyceraldehyde-3-phosphate dehydrogenase) as the internal control. The results are expressed relative to the untreated control value and are the mean ± S.D. for three independent experiments. *: p <0.05 compared to the untreated control; **: p <0.005 compared to the untreated control.

UCP2 mRNA levels were increased by treatment with 100 μM EPA or 50 μM DHA, the increase with EPA being greater (Figure 2).

### Pretreatment with EPA or DHA inhibits the DOX-induced decrease in UCP2 expression

**Figure 3:**
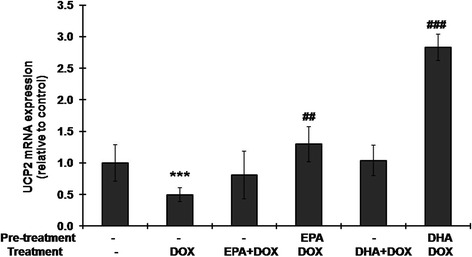
**Pretreatment with EPA or DHA prevents the doxorubicin-induced decrease in UCP2 mRNA levels.** H9C2 cells were (i) left untreated or (ii) treated with 100 μM EPA or 50 μM DHA for 24 h, then treated with 1 μM doxorubicin for 24 h or (iii) treated with 1 μM doxorubicin (DOX) in the absence or presence of 100 μM EPA or 50 μM DHA for 24 h, then UCP2 mRNA levels were measured by reverse transcription and quantitative real-time PCR, with GAPDH (glyceraldehyde-3-phosphate dehydrogenase) as the internal control. The results are expressed relative to the untreated control value and are the mean ± S.D. for three independent experiments. ***: p <0005 compared to the untreated control; ^##^, ^# # #^: p <0.01 and p <0.001 compared to doxorubicin treatment alone, respectively.

To examine whether the DOX-induced decrease in UCP2 mRNA levels could be inhibited by pretreatment or cotreatment with EPA or DHA, H9C2 cells were either pretreated for 24 h with 100 μM EPA or 50 μM DHA followed by 1 μM DOX treatment for 24 h in the continued presence of the fatty acid or were cotreated with 1 μM DOX and either 100 μM EPA or 50 μM DHA for 24 h, then UCP2 mRNA levels were measured. As shown in Figure 3, EPA pretreatment prevented the DOX-induced decrease in UCP2 mRNA expression and DHA pretreatment before DOX treatment results in a large increase in UCP2 mRNA levels, whereas cotreatment with EPA or DHA and DOX had no significant effect compared to treatment with DOX alone.

**Figure 4:**
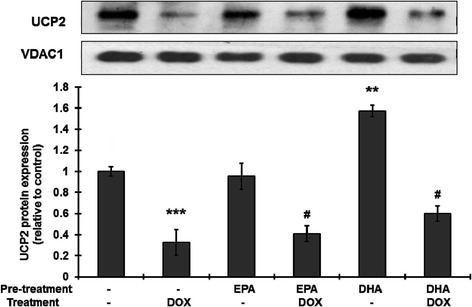
**Pretreatment with EPA or DHA prevents the doxorubicin-induced decrease in UCP2 protein expression.** H9C2 cells were left untreated or were treated with 50 μM DHA or 100 μM EPA for 24 h, then were left untreated or wre treated with 1 μM doxorubicin (DOX) in the continued presence of the fatty acid for 24 h. After treatment, the cells were harvested and proteins extracted for Western blotting using antibody against UCP2 with ß-actin as the internal control. The bars are the quantitative density analysis expressed as the relative density compared to that in the untreated control and are the mean ± S.D. for three separate experiments. **, ***: p <001 and p <0.005 compared to the untreated control, respectively; ^#^: p <0.05 compared to doxorubicin treatment.

In order to examine the effect on UCP2 protein expression, H9C2 cells were left untreated or were pretreated for 24 h with 100 μM EPA or 50 μM DHA, then were incubated with or without 1 μM DOX treatment for 24 h in the continued presence of the fatty acid and proteins were extracted for Western blotting. Figure 4 shows that, in accordance with the mRNA result (Figure 2), EPA treatment significantly increased UCP2 protein expression as compared to the control. As expected, DOX treatment markedly decreased UCP2 protein expression and this effect was significantly inhibited by pretreatment with EPA or DHA. The large increase in UCP2 mRNA levels seen with DHA pretreatment before DOX treatment (Figure 3) was not reflected in the protein levels (Figure 4), suggesting a less effective translocation of UCP2 protein to the mitochondrial membrane.

### Pretreatment with EPA or DHA inhibits the DOX-induced ROS production, mitochondrial membrane potential change and cell death

**Figure 5:**
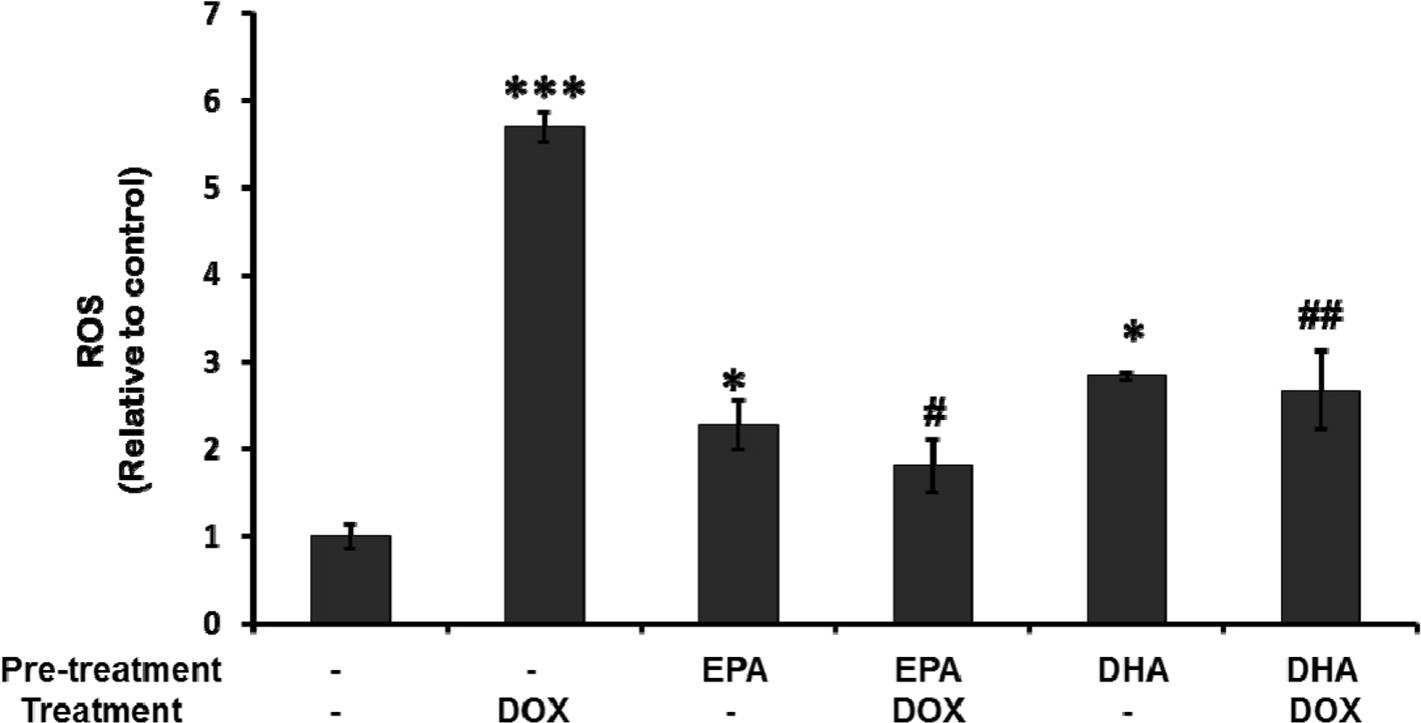
**Pretreatment with EPA or DHA prevents doxorubicin-induced reactive oxygen species (ROS) production.** H9C2 cells were treated as in Figure 4, then intracellular ROS levels were measured using H_2_DCF-DA dye and flow cytometry. The results are expressed as the mean ± S.D. for three separate experiments. *, ***: p <005 and p <0.005 compared to the untreated control, respectively; ^#^, ^# #^: p <0.05 and P <0.01 compared to doxorubicin treatment, respectively.

**Figure 6:**
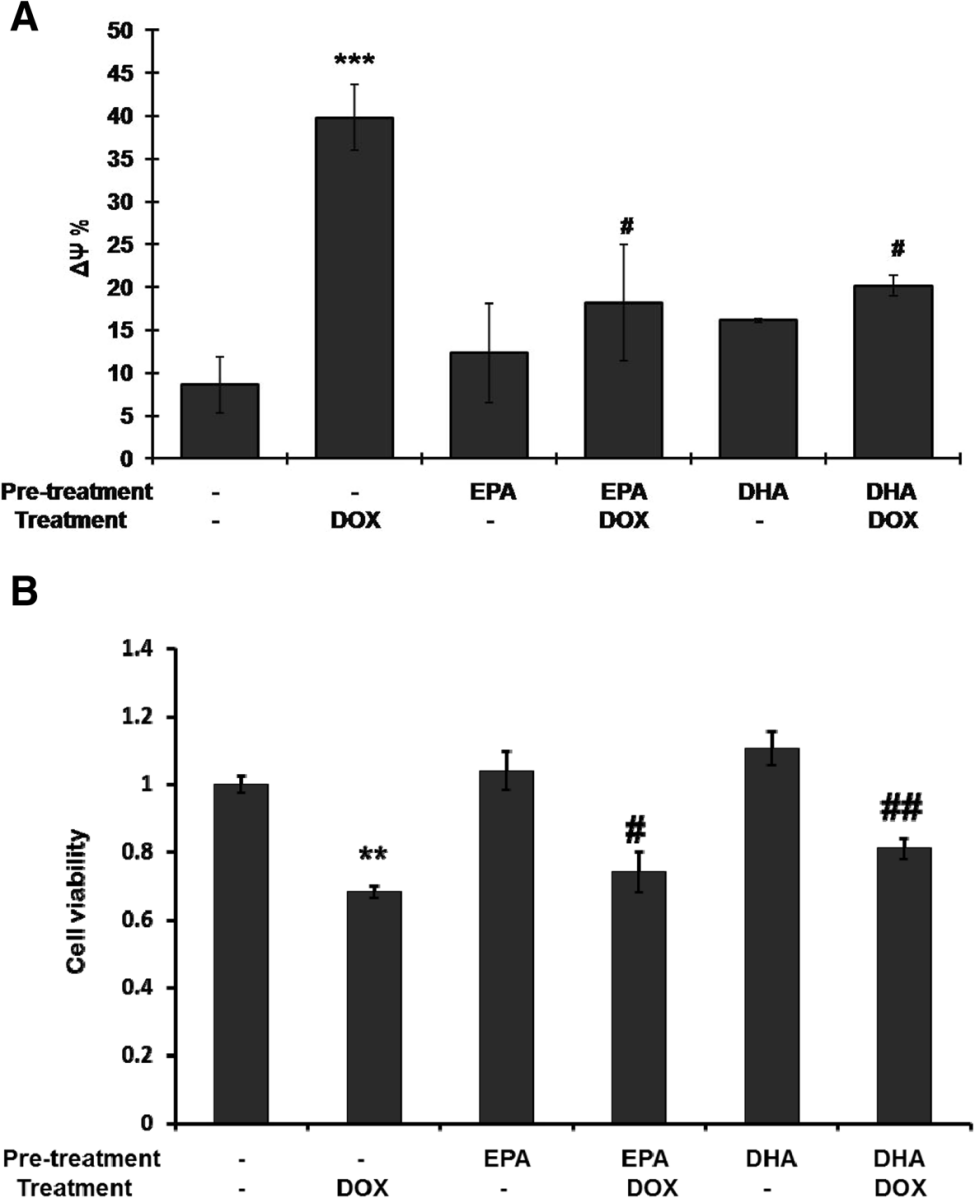
**Pretreatment with EPA or DHA prevents the doxorubicin-induced change in the mitochondrial membrane potential (∆ψ) (A) and cell viability (B).** H9C2 cells were treated as in Figures 4 and 5, then the ∆ψ was measured using JC-1 dye and flow cytometry. The results are expressed as the mean ± S.D. for three separate experiments. **, ***: p <0.01 and p <0005 compared to the untreated; ^#^, ^##^: p <0.05 and p <0.01 compared to doxorubicin treatment.

Several studies have ascribed the cardiotoxicity of DOX to the induction of ROS [3] and mitochondria collapse [8]. We therefore examined whether EPA or DHA pretreatment of cells subsequently treated with DOX had an effect on ROS and/or the mitochondrial membrane potential. Figure 5 shows that DOX treatment dramatically increased ROS production and that, although treatment with either EPA or DHA alone resulted in a significant increase in ROS as compared to the untreated control, pretreatment with EPA or DHA reduced the DOX-induced increase. Figure 6A shows that DOX treatment results in a large decrease in the mitochondrial membrane potential (∆ψ) and that this effect was reduced by pretreatment with EPA or DHA. A concordant result in cell viability was found. DOX-induced cell death was prevented by the pretreatment with EPA or DHA (Figure 6B).

## Discussion

In this study, we found that DOX decreased UCP2 mRNA and protein levels in H9C2 cells and that this was related to cell viability. Pretreatment with 100 μM EPA or 50 μM DHA for 24 h resulted in the maximal concentration of EPA or DHA, respectively, in mitochondrial fatty acid composition and prevented the DOX-induced reduction in UCP2 expression. However, this effect was not found with cotreatment with DOX and either EPA or DHA. Although EPA or DHA pretreatment alone increased ROS production, both effectively inhibited subsequent DOX-induced ROS production and significantly inhibited the DOX-induced collapse in the mitochondrial membrane potential (∆ψ). These findings show that EPA or DHA pretreatment prevented the reduction in UCP2 expression induced by DOX that might contribute to the cardiomyocyte toxicity of DOX.

DOX, a quinine-containing drug, can be converted to the semiquinone form by the transfer of one electron. The DOX semiquinone can subsequently transfer an electron to the oxygen molecule (O_2_) to form the superoxide anion radical (O_2_^−^) [3]. There is evidence that DOX can generate ROS by two distinct mechanisms as it enters the cell. The first involves DOX forming a complex with iron (III) and transferring one electron to the iron to generate an iron (II)-DOX free radical complex, which can then reduce oxygen and hydrogen to superoxide anion radical and hydroxyl radical [25]. The second involves DOX generating ROS through redox cycling, which is catalyzed by a number of NAD(P)H oxidoreductases [3,26]. It is believed that, at the subcellular level, mitochondria are the primary target of DOX-induced cardiotoxicity, since heart tissue is rich in mitochondria. Previous study has indicated that the accumulation of redox-active DOX in these organelles [3,27] would enhance mitochondrial production of ROS, resulting in interference with mitochondrial respiratory function. In accordance with these findings, this study shows a marked increase in ROS production in H9C2 cells treated with DOX for 24 h.

UCPs are members of the anion carrier protein family located in the inner mitochondrial membrane [17] and do not transport protons in the absence of specific activators, but do transport protons and increase the net proton conductance of mitochondria in the presence of specific activators [28-30], including ROS and reactive alkenals, such as hydroxynonenal, which is produced by peroxidation of membrane phospholipids [31]. In the present study, we found that, although DOX induced a marked increase in ROS in H9C2 cells, UCP2 levels were not increased and were even lower than basal levels, which did not lessen the oxidative stress and resulted in mitochondrial depolarization, leading to cell death. In contrast, pretreatment with EPA or DHA inhibited the DOX-induced decrease in UCP2 expression. UCP2 mediates mild uncoupling and thus mitigates ROS production.

It is still unclear why and how UCP2 expression is decreased dramatically by DOX; changes in the composition of cardiolipin could be one of the possible mechanisms. Cardiolipin, the major phospholipid in the mitochondrial membrane, participates in several important processes in mitochondria, including oxidative phosphorylation, apoptosis and the assembly and functioning of mitochondrial membrane proteins [7]. Altering the membrane phospholipid composition changes the normal mitochondrial functions. A previous study showed that DOX interacted with cardiolipin, modulated mitochondrial membrane lipid composition, and therefore caused mitochondrial dysfunctions [7]. Similarly, n-3 PUFAs affect the compositions of cardiolipin as well. Dietary supplementation of n-3 PUFAs increases the level of DHA in the cardiolipin, decreases mitochondrial depolarization and therefore increases cell survival [11]. We found that pretreatment with n-3 PUFAs prevented DOX-induced reduction in UCP2 expression, however, simultaneous treatment with n-3 PUFAs and DOX did not. Pretreatment with n-3 PUFAs increased their incorporation into the mitochondrial membrane and modulated cardiolipin composition in advance, while in the simultaneous treatment, the n-3 PUFAs had less effect on mitochondrial composition due to their competition with DOX in modulating lipid composition at the same time. Taken together, we propose that the protective effect of n-3 PUFAs might be due to the incorporation of n-3 PUFAs into cell membrane phospholipids and cardiolipin [10,11] rather than to a direct effect on UCP2, thus altering membrane fluidity, modulating cell signaling [32], and enhancing free radical production [33]. Further experiments are needed to explore the transcriptional events and target genes related to UCPs that are affected by DOX and n-3 PUFAs.

N-3 PUFAs have diverse effects on cardiomyocytes and cancer cells. In cardiomyocytes, n-3 PUFAs protect cells from cardiac injury via multiple molecular mechanisms including anti-inflammation, altering the membrane composition and fluidity, regulating gene expression, converting to bioactive metabolites and maintaining mitochondrial functions [10,11]. As anti-cancer agent, in contrast, n-3 PUFAs are associated with the properties of anti-inflammation, anti-proliferation, proapoptosis, anti-metasis, anti-invasion, and anti-angiogenesis [14]. It is evident that n-3 PUFAs induce apoptosis in cancer cells by triggering excessive ROS production and inducing autophagy [34,35]. Having more unsaturated double bonds and *bis*-allylic hydrogens than monounsaturated fatty acid, n-3 PUFAs are much more prone to spontaneous oxidation and result in an increase in ROS production [36]. Compared to normal cells, cancer cells have higher levels of mitochondrial ROS generation for cellular proliferation and tumorigenesis, the ROS products further attack n-3 PUFAs and cause more lipid peroxidation and lead to apoptosis in cancer cells [14]. In normal cells, lipid peroxides are the major ROS products generated by n-3 PUFAs [37-39], whereas neither oxidatively modified protein nor oxidized LDL is induced by n-3 PUFA supplementation [39,40]. In addition, accumulating evidences show that oxidative modification of n-3 PUFAs is biologically active and benefits the cardiac functions [41-43]. Consequently, although our data show a small but statistically significant increase in ROS production induced by n-3 PUFAs, the clinical relevance of this change is questionable. It is evident that n-3 PUFA pretreatment reduces DOX-induced ROS production. However, the present data could not clarify whether n-3 PUFA reduces ROS generation via UCP2 or not. UCP2 overexpression and a short interfering RNA system are required to further elucidate the related mechanism.

It has been reported that the amount of DOX-induced ROS production is positively correlated with the membrane unsaturation index of fatty acids; these lipid peroxides have been implicated in the cytotoxic process and the increase in drug efficacy in cancer cells [20,44,45]. Our results showed that pretreatment with EPA or DHA substantially increased the unsaturation index of mitochondria and enhanced ROS production in H9C2 cells in the absence of DOX. However, when the cells were treated with DOX, ROS production was not significantly changed in the EPA or DHA pretreated group, but was markedly increased in the non-pretreated group. Accordingly, we propose that myocytes and cancer cells may have different mechanisms for responding to DOX-induced toxicity, and whether UCP expression is upregulated or not may determine whether n-3 PUFAs cause an improvement or worsening of DOX-induced oxidative stress.

## Conclusion

EPA or DHA pre-treatment inhibits the DOX-induced decrease in UCP2 expression, increase in ROS production, and subsequent mitochondrial membrane potential change that contribute to the cardiotoxicity of DOX. This study highlights the possibility that pretreatment with n-3 PUFAs might be useful in reducing DOX-induced oxidative stress in cardiomyocytes, and provides clues for studying the role of UCPs in this mechanism. However, the present cell model did not provide solid evidences to verify all the protective mechanisms of n-3 PUFA on DOX-induced cytotoxicity. In the future, conducting a gain-of-function/loss-of-function assay, especially via a transgenic animal model, would be preferable for obtaining information on gene function and regulation by n-3 PUFAs as well as on DOX-induced cardiomyocyte cytotoxicity.

## Additional file

Additional file 1:
**Dose-dependent cellular response in n-3 PUFA treated H9C2 cells.**
