# Supplementary material for: N-3 polyunsaturated fatty acids decrease levels of doxorubicin-induced reactive oxygen species in cardiomyocytes -- involvement of uncoupling protein UCP2
Source: J Biomed Sci. 2014 Nov 18;21(1):101. doi: 10.1186/s12929-014-0101-3 (PMC4237738; doi:10.1186/s12929-014-0101-3)
Supplement: Additional file 1: — Dose-dependent cellular response in n-3 PUFA treated H9C2 cells. [file 12929_2014_101_MOESM1_ESM.docx]

Additional file 1. Dose-dependent cellular response in n-3 PUFA treated H9C2 cells

|  | 0 uM | 50 uM | 100 uM |
| --- | --- | --- | --- |
| EPA treatment for 24 hr | | | |
| EPA % in total cell lysate | 1.00 | 5.83 | 6.42 |
| EPA % in mitochondrial membrane | 0.77 | 5.13 | 5.74 |
| EPA treatment for 48 hr |  |  |  |
| EPA % in total cell lysate | 1.08 | 5.56 | 10.49 |
| EPA % in mitochondrial membrane | 0.59 | 3.56 | 5.43 |
|  |  |  |  |
| DHA treatment for 24 hr |  |  |  |
| DHA % in total cell lysate | 3.13 | 13.54 | 22.96 |
| DHA % in mitochondrial membrane | 4.53 | 10.12 | 10.04 |
| DHA treatment for 48 hr |  |  |  |
| DHA % in total cell lysate | 2.67 | 14.19 | 18.41 |
| DHA % in mitochondrial membrane | 3.68 | 10.16 | 10.52 |
